# A touchscreen-based paradigm to measure visual pattern separation and pattern completion in mice

**DOI:** 10.3389/fnins.2022.947742

**Published:** 2022-08-24

**Authors:** Hao Wang, Na Sun, Xinyue Wang, Jinyuan Han, Yongxiang Zhang, Yan Huang, Wenxia Zhou

**Affiliations:** ^1^Beijing Institute of Pharmacology and Toxicology, Beijing, China; ^2^State Key Laboratory of Toxicology and Medical Countermeasures, Beijing Institute of Pharmacology and Toxicology, Beijing, China

**Keywords:** memory accuracy, pattern separation, pattern completion, rodents, pairwise discrimination task, touchscreen

## Abstract

Memory accuracy involves two major processes: pattern separation and pattern completion. Pattern separation refers to the ability to reduce overlap among similar inputs to avoid interference, and pattern completion refers to the ability to retrieve the whole information from partial or degraded cues. Impairments in pattern separation/pattern completion contribute to cognitive deficits in several diseases of the nervous system. Therefore, it is better to evaluate both pattern separation and pattern completion in one apparatus. However, few tools are available to assess pattern separation and pattern completion within the same apparatus for rodents. In this study, we designed a series of images with varying degrees of similarity to the correct image to evaluate pattern separation and pattern completion. First, mice were trained to discriminate between two totally different images, and once the correct percentage reached above 77% for two consecutive days, the images with different degrees of similarity were used to measure pattern separation and pattern completion. The results showed the mice performed progressively worse from S0 to S4 (increasing similarity) when discriminating similar images in pattern separation, and the mice performed progressively worse from C0 to C4 (decreasing cues information) when recalling the correct image according to partial cues in pattern completion, implying a good image similarity-dependent manner for memory accuracy evaluation. In sum, we designed a convenient, effective paradigm to evaluate pattern separation and pattern completion based on a touchscreen pairwise discrimination task, which may provide a new method for the studies of the effects and mechanisms of memory accuracy enhancing drugs.

## Introduction

The accuracy of episodic memory contains temporal and spatial information of events (Tulving, 1972), and its underlying neurobiological mechanisms are based on two processes, namely pattern separation and pattern completion (Ngo et al., 2021). Pattern separation is a computational process of transforming similar memory engrams into different or orthogonal ones, and pattern completion is a computational process of retrieving the previously stored memory in response to partial or degraded cues (Hunsaker and Kesner, 2013). The accuracy of episodic memory is significant in daily life, such as in tests or interviews. Moreover, it was found that impairments in pattern separation/pattern completion are concerned with many diseases, such as amnestic mild cognitive impairment (Yassa et al., 2010; Lalani et al., 2021), Alzheimer’s disease (Lee et al., 2020; Parizkova et al., 2020), schizophrenia (Das et al., 2014; Martinelli and Shergill, 2015), and post-traumatic stress disorder (Iris et al., 2017; Bernstein et al., 2020). Additionally, pattern separation (Nitschke et al., 2020) and pattern completion (Vieweg et al., 2015) are highly susceptible to stress, which may show impaired memory accuracy.

The motivations of currently used behavioral paradigms for pattern separation evaluation mainly include three kinds: neutral, negative, and positive paradigms (Zheng et al., 2019). Neutral paradigms include the temporal ordering task (Kannangara et al., 2015), the spontaneous location recognition task (Reichelt et al., 2021), object pattern separation task (van Goethem et al., 2018; Heckman et al., 2020), etc. The rationales of the neutral paradigms mainly depend on the natural curiosity of animals for new objects or locations, and such paradigms are easily operated, but the task motivation is vulnerable to disturbance, such as stress. Stress may affect motivation in some behavioral tests, and the disturbed motivation may further confuse behavior analysis. For example, bright light is a stressor for rodents; mice maintained in brighter light exhibited spatial memory impairment in the Y maze (Shang et al., 2021); and light-intensity-dependent disruptive effects on the object and odor recognition memory task were also reported (Hasan et al., 2021). Contextual fear discrimination (Kheirbek et al., 2012) and its adapted versions are typically negative paradigms. Some researchers argued that a single foot-shock could cause long-lasting hyperactivity (Piérard et al., 2009), which is possibly associated with deficits in pattern separation. The location discrimination task, the trial-unique delayed non-matching-to-location task based on the touchscreen (Oomen et al., 2013), and the radial-arm maze (Clelland et al., 2009) belong to positive paradigms due to their sufficient motivation. These three paradigms are commonly used to measure spatial pattern separation, not suitable for object pattern separation. When it comes to pattern completion paradigm, there are limited paradigms available for rodents, such as the Morris water maze with four different-sized visual cues in four quadrants (Fellini et al., 2009; Li et al., 2010), home-made cue-based preference box (Kesner et al., 2016). These paradigms are based on the definition of pattern completion and are user-friendly, but difficult to manipulate the cue similarities accurately. And so far, there is no paradigm that can evaluate both pattern separation and pattern completion within the same apparatus.

The Skinner box, invented by the behaviorist Meehl (1992), is mainly used in animal experiments related to psychology. Nowadays, the touchscreen, a new version of the Skinner box, is not only used in the psychological study but also in nervous system diseases (Palmer et al., 2021). Sufficient motivation driven by food restriction, enclosed environment, stress-free from bright light or smell, and other advantages enable paradigms based on touchscreen technology to be excellent cognitive testing tools. Pairwise discrimination task, one of the typical touchscreen paradigms, designed to examine the ability to identify two different images (Bussey et al., 1994), is simple to operate and is able to be modified to achieve our purpose. In this study, we established a paradigm aiming at measuring pattern separation and pattern completion based on a touchscreen pairwise discrimination task.

## Materials and methods

### Animals

Thirty-two male C57BL/6J (6 weeks old) mice were purchased from Beijing SPF Biotechnology Company [license number: SCSK (Beijing) 2016-0002]. Mice were group housed on a 12-h light–dark cycle (lights on from 7:00 to 19:00) at 23 ± 1°C and 50 ± 5% humidity and got free access to food and water during 7 days of acclimation. For animals’ welfare, paper tubes, nesting material, ping-pong balls, and 3D-printed polygons (cube, sphere, regular dodecahedron, and regular icosahedron) were placed into cages in turn to create a rich environment for the mice. Prior to the training, mice were restricted to food for 3 days (1 h a day) to maintain their body weight at 85–90% of their free-feeding weight (free-feeding: 19.0–22.0 g, food deprivation: 16.7–19.4 g). Once the experiment began, the mice were restricted to food for 60–120 min a day to maintain their task motivation. All experiments were approved by the Institute of Animal Care and Use Committee (IACUC) of the National Beijing Center for Drug Safety Evaluation and Research (NBCDSER).

### Touchscreen operant conditioning

The whole touchscreen apparatus has four operating chambers (Campden Instruments Ltd., United Kingdom); each of which is equipped with grid flooring, overhead light, touchscreen with an infrared frame, reward dispenser, food tray, 2-window mask (7.0 cm × 7.5 cm), and camera above the chamber (Lim et al., 2019). The running schedule is supported by Whisker Server. The mask, touchscreen, grid floor, and food tray were cleaned with 37% alcohol between animals. At the end of daily testing, all reward lines were flushed with clean water and then pumped dry to prevent blockages. The tray under the chamber served to collect fecal boli and was supposed to be cleaned daily. Results from the experiments can be analyzed by the ABET II Touch software (Lafayette Instruments Co., Lafayette, IN, United States).

### Pairwise discrimination task training

**FIGURE 1:**
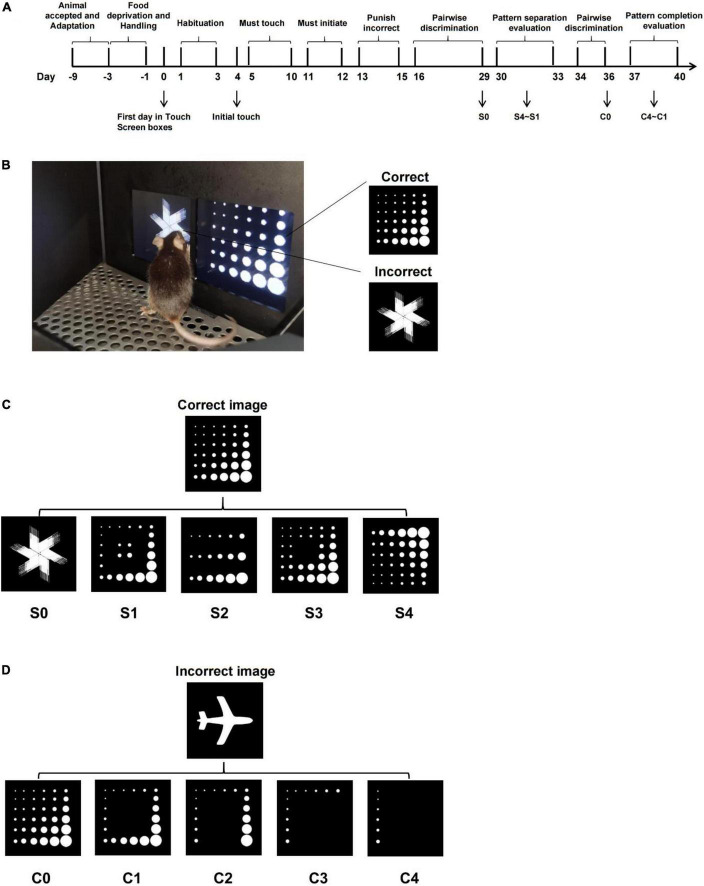
Schematic representation of adapted paradigms for pattern separation and pattern completion based on pairwise discrimination task. **(A)** Training flowchart of the pairwise discrimination task. **(B)** Schematic representation of touchscreen (correct image “Marble” is on the right side of the screen; incorrect image “Fan” is on the left side of the screen). **(C)** Schematic representation of pattern separation paradigm of varying difficulty (“S” means separation; the number behind S represents the difficulty index, the higher the number, the more difficult it is). **(D)** Schematic representation of pattern completion paradigm of varying difficulty (“C” means completion; the number behind C represents the difficulty index; the higher the number, the more difficult it is).

All the training processes were carried out according to the Pairwise (Visual) Discrimination Task Instruction Manual (Campden Instruments Ltd., United Kingdom and Lafayette Instrument Co.) and Horner et al. (2013). Food pellet rewards were believed to limit the number of trials per experiment; therefore, we used a liquid reward (strawberry milkshake) in this study instead. The strawberry milkshake was made of 5 g condensed milk (purchased from Nestle) and 0.5 g sucrose dissolved in 45 ml water. Images used in stage 2 to stage 5 consisted of various shapes, and images used in stage 6 were named “Marble” and “Fan.” The whole training process consists of six stages (Figure 1A), as explained below:

Stage 1 (Habituation): Mice were habituated to chambers for 20, 40, and 40 min, respectively, for three consecutive days until the training day ended. In the beginning, the food tray was primed with rewards (150 μl, 6,000 ms) with a tone played and a food tray light on. Once the mouse consumed the reward, the food light was turned off. The food tray light was turned on at 10 s delay, and the reward (7 μl, 280 ms) was then delivered with a tone played. Data were excluded when there was a deviation from mean ± 3 × standard deviation (SD).

Stage 2 (Initial Touch): One image was presented on one side of the screen (within the windows of the mask), and the other side of the screen was left blank. If the mouse touched the screen while the image was displaying, the image was removed followed by a tone, and 3 × food (21 μl, 280 ms) was delivered immediately. If the mouse touched the blank side of the screen, only 1 × food (7 μl, 280 ms) was delivered immediately. The criterion was to complete 30 trials in 60 min.

Stage 3 (Must Touch): An image was displayed randomly on one side of the screen at a time, the mouse must touch the image to elicit a tone/food response. There was no response if the mouse touched the blank side of the screen. The criterion was to complete 30 trials in 60 min.

Stage 4 (Must Initiate): In the beginning, free delivery of food was offered, and the tray light was turned on. The mouse must nose poke and then exit the food tray before an image was displayed randomly on the screen. Other operations and criteria were the same as those in stage 3.

Stage 5 (Punish Incorrect): When the mouse touched the image on the screen, the reward was delivered as usual. Once the mouse touched the blank side of the screen, the house light would be turned on for a time-out period (5 s), and no reward was delivered. The criterion was to complete 23/30 trials correctly (77%) in 60 min for 2 days in a row.

Stage 6 (Discrimination Training): A trial began with the presentation of two different images on the screen, image “Marble” was programmed as being correct and the image “Fan” as being incorrect. When the mouse touched the image “Marble,” the reward was delivered as usual. If the mouse’s nose poked the image “Fan,” it would be punished by the house light. The criterion was to complete 23/30 trials correctly (77%) in 60 min for 2 days in a row (Figure 1B). Once the mice meet the criterion of the pairwise discrimination task, paradigm adaptation experiments could be carried out.

### Establishment of pattern separation and pattern completion paradigm

Research into pattern separation and pattern completion could be measured on the condition that two features could be fulfilled: on the one hand, interferences/cues among stimuli were parametrically altered; on the other hand, behavioral responses scale with interferences/cues. Pattern separation and pattern completion paradigms were developed based on the pairwise discrimination task as described above, with modifications of the correct or incorrect images.^1^

In terms of the pattern separation evaluation paradigm, to obtain the reward, the mice must discriminate the correct image from an incorrect image (varying degrees of similarity to the correct image). The correct image “Marble” stayed unchanged, whereas the incorrect image was transformed from the correct one by covering (S1–S3) or rotating (S4, Figure 1C). From S1 to S3, the similarity was controlled by covering a different number of marbles within image “Marble,” and similarity from S1 to S4 was progressively increased, which placed a higher demand on pattern separation. A trial started with the presentation of two images on the screen, image “Marble” was programmed as being correct, and image adapted from “Marble” (S1–S4) was programmed as being incorrect. Whether the correct image appeared on the left or right was determined pseudo-randomly, such that the image would not be displayed on the same side more than three times in a row. The mouse must nose poke the correct image to get rewarded, which was accompanied by illumination of the tray light and a tone, or it would get punished by house light for 5 s. Collecting the reward turns off the tray light and starts the intertrial interval (ITI), the tray light was again illuminated after the ITI period (20 s). The mouse must nose poke and exit the food tray to initiate the next trial and elicit the images to be displayed again. The end of 60 min or 30 trials completed (whichever happens first) indicates the end of the experiment. The evaluation sequence of pattern separation was conducted from difficult to easy (from S4 to S1). The incorrect image changed daily, and the pattern separation paradigm was established for a total of 4 days.

In terms of the pattern completion evaluation paradigm, to avoid the mice from making a choice *via* the exclusion method, a new image “Plane” was selected as the incorrect one; the adapted correct image was a part of the original correct image “Marble” (C1–C4, Figure 1D). From C1 to C4, cue information was manipulated by decreasing the number of marbles within the image “Marble.” The cues information from C1 to C4 was progressively decreasing, which place a higher demand on pattern completion. The mice are required to recognize the correct image by a given amount of cue (C1–C4) for a reward. The experiment process is the same as in the pattern separation evaluation described earlier.

Each experiment contained 30 trials; there were 3 blocks in each experiment as every 10 trials were divided into a block automatically. Correct percentage, the total number of correction trials (if the mouse nose poked the incorrect image, then it was given the opportunity to complete a “correction trial,” which would ensue until the correct image was chosen. “Number of correction trial” was the number of trials before the mouse made the correct choice) and the time required to complete 30 trials and these indices in each block were adopted to index the behavioral outcome of pattern separation and pattern completion. Besides this, the number of trials completed in 60 min was also chosen to index the task difficulty. The indices illustrated above are applicable to both pattern separation and pattern completion.

### Statistical analysis

Data were expressed as mean values ± SD of the mean. The statistical analyses were conducted with GraphPad Prism 8.0.1 software. One-sample *t*-statistics were used to assess whether the correct percentage for each test separately differed significantly from the chance level (chance level = 50%). The one-way ANOVA was used to compare images of different difficulties; Dunnett’s *post hoc* analyses were carried out once the overall ANOVA was significant. A two-way repeated-measures ANOVA (S0–S4/C0–C4 and Block 1–Block 3) was used to compare the performance in each block. The *p*-value of <0.05 was considered statistically significant.

## Results

### Training results of the pairwise discrimination task

**FIGURE 2:**
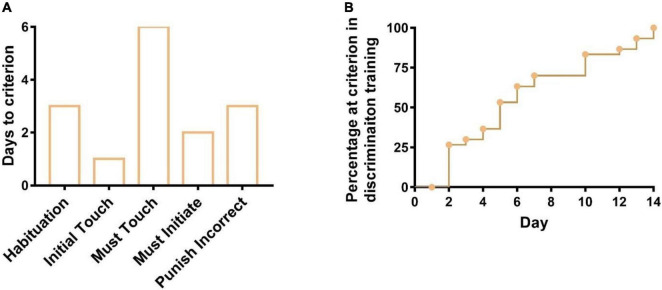
Days to meet the criterion for six training stages during the pairwise discrimination task. **(A)** Days to meet the criterion from stage 1 to stage 5 and **(B)** days to meet the criterion in stage 6 (discrimination training).

There are six stages in pairwise discrimination tasks. During stage 1 (Habituation), after 3 days (1st to 3rd days) of training, 31 out of 32 mice completed an average of 147 trials on the 3rd day, one mouse was excluded from the latter training as less than 10 trials completed, indicating 31 mice have learned how to get rewarded (milkshake) by cues (tray light and a tone). During stage 2 (Initial Touch), all the 31 mice were able to complete 30 trials in 60 min after 1 day (4th day) of training, indicating they learned that touching images on the screen could elicit more rewards. During stage 3 (Must Touch), all the 31 mice were able to complete 30 trials in 60 min after 6 days (5th–10th days) of training, indicating they have learned to get rewarded by touching the image on the screen. During stage 4 (Must Initiate), all the 31 mice could complete 30 trials in 60 min after 2 days (11th and 12th days) of training, indicating all mice have learned to initiate the trial by nose poking and exiting the reward tray themselves. During stage 5 (Punish Incorrect), after 3 days (13th–15th days) of training, the correct percentage of 28 out of 31 mice could achieve 77% in 60 min when they were trained not to touch the blank side of the screen, whereas the correct percentage of the 3 out of 31 mice were less than but close to 77% (qualified line). As a result, all 31 mice were given chances at the last training stage (Figure 2A). During stage 6 (Discrimination Training), all the 31 mice could achieve 77% of the correct percentage for 2 consecutive days after 14 days (16th–29th days) of training, implying 31 mice had learned the pairwise discrimination task and were ready for further experiments (Figure 2B).

### Establishment of paradigm to measure pattern separation

**FIGURE 3:**
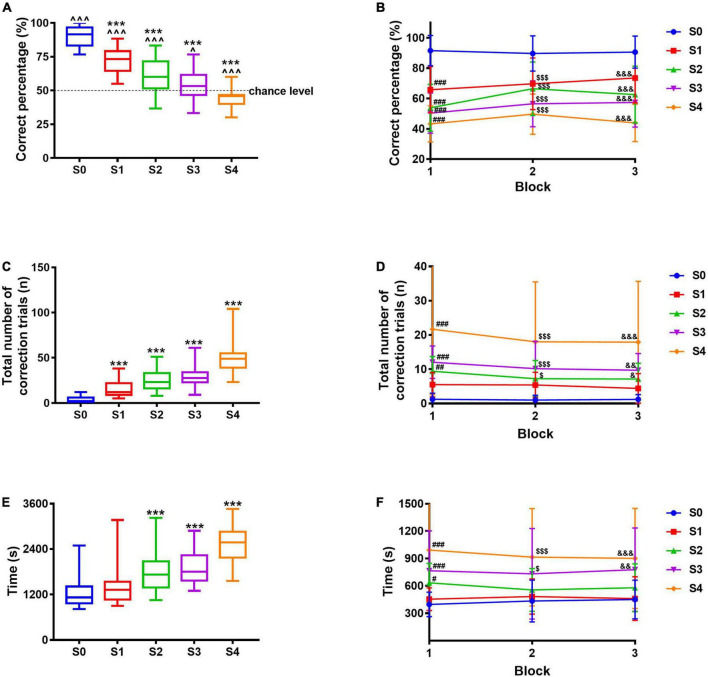
Pattern separation evaluation paradigm using different images. **(A)** Correct percentage. **(B)** Correct percentage in each block. **(C)** The total number of correction trials. **(D)** The total number of correction trials in each block. **(E)** The total time required to complete 30 trials. **(F)** Time required to complete every 10 trials. Data are presented as the mean ± SD (*n* = 30–31). ^*p* < 0.05 and ^^^*p* < 0.001 compared with chance level, one-sample *t*-test. ***p < 0.001 compared with the S0 group, one-way ANOVA followed by the *post hoc* Dunnett’s *t*-test. ^#^*p* < 0.05, ^##^*p* < 0.01, and ^###^*p* < 0.001 compared with the S0 group during Block 1; ^$^*p* < 0.05 and ^$$$^*p* < 0.001 compared with the S0 group during Block 2; ^&^*p* < 0.05, ^&&^*p* < 0.01, and ^&&&^*p* < 0.001 compared with the S0 group during Block 3, two-way repeated-measures ANOVA followed by Tukey’s *t*-test.

Based on the pairwise discrimination task, the correct image “Marble” and the incorrect image, that is, adapted “Marble,” were used to measure pattern separation. The correct percentage of S0, S1, S2, S3, and S4 was significantly different from the chance level (50%, *p* < 0.001, *p* < 0.001, *p* < 0.001, *p* = 0.0206, and *p* = 0.0003, respectively), indicating the mice did not make choice randomly. With images varying from S0 to S4 (from easy to difficult), the correct percentage ranged from 90.6% (S0) to 45.4% (S4), indicating a gradual decrease (Figure 3A), so were these in each block (Figure 3B); compared with S0, the correct percentage decreased significantly for S1, S2, S3, and S4 (*p* < 0.001, comprehensively). The total number of correction trials ranged from 3.5 times (S0) to 49.5 times (S4, Figure 3C), indicating a gradual increase, so were these in each block (Figure 3D); compared with S0, the number of correction trials increased significantly for S1, S2, S3, and S4 (*F*_4_,_149_ = 72.67, *p* < 0.001, comprehensively). Likewise, the total time required to complete 30 trials ranged from 1245.1 s (S0) to 2514.3 s (S4, Figure 3E), indicating a gradual increase, so were these in each block (Figure 3F), the total time required to complete 30 trials of S2, S3, and S4 increased significantly compared with that of S0 (*F*_4_,_149_ = 35.77, *p* < 0.001, comprehensively). With images varying from easy to difficult, three mice failed to complete 30 trials in 60 min during S3 and S4; although most of the mice succeeded, indicating the difficulty represented by the five images was suitable for this test. In consequence, the images adopted in this study showed a good difficulty gradient, implying a successful pattern separation evaluation paradigm.

### Establishment of paradigm to measure pattern completion

**FIGURE 4:**
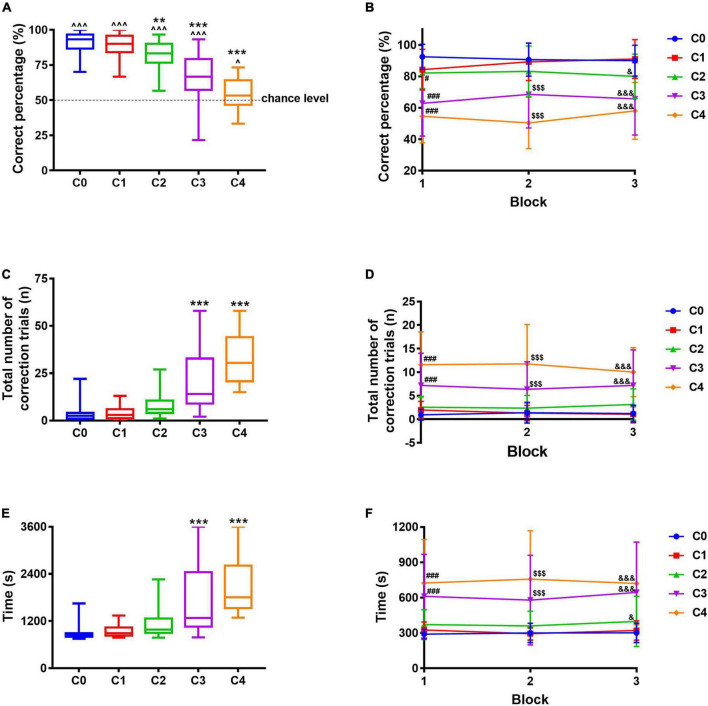
Pattern completion evaluation paradigm using different images. **(A)** Correct percentage. **(B)** Correct percentage in each block. **(C)** The total number of correction trials. **(D)** The total number of correction trials in each block. **(E)** The total time required to complete 30 trials. **(F)** Time required to complete every 10 trials. Data are presented as the mean ± SD (*n* = 26–28). ^*p* < 0.05 and ^^^*p* < 0.001 compared with chance level, one-sample *t*-test. **p < 0.01 and ***p < 0.001 compared with the C0 group, one-way ANOVA followed by *post hoc* Dunnett’s *t*-test. ^#^*p* < 0.05 and ^###^*p* < 0.001 compared with the C0 group during Block 1; ^$$$^*p* < 0.001 compared with the C0 group during Block 2; ^&^*p* < 0.05 and ^&&&^*p* < 0.001 compared with the C0 group during Block 3, two-way repeated-measures ANOVA followed by Tukey’s *t*-test.

Based on the pairwise discrimination task, the adapted correct image “Marble” and the incorrect image “Plane” were used to measure pattern completion. The correct percentage of C0, C1, C2, C3, and C4 was significantly different from the chance level (*p* < 0.001 for C0–C3, *p* = 0.0315 for C4), indicating the mice did not make the choices randomly. With images varying from C0 to C4 (from easy to difficult), the correct percentage ranged from 91.1% (C0) to 54.7% (C4), suggesting a gradual decrease (Figure 4A), so were these in each block (Figure 4B); compared with C0, the correct percentage decreased significantly for C2, C3, and C4 (*F*_4_,_149_ = 49.54, *p* = 0.0027, *p* < 0.001, and *p* < 0.001). The total number of correction trials ranged from 3.5 times (C0) to 31.9 times (C4, Figure 4C), suggesting a gradual increase, so were these in each block (Figure 4D); compared with C0, the correction trials increased significantly for C3 and C4 (*F*_4_,_149_ = 42.72, *p* < 0.001 and *p* < 0.001). Likewise, the total time required to complete 30 trials ranged from 892.3 s (C0) to 2137.4 s (C4, Figure 4E), suggesting a gradual increase, so were these in each block (Figure 4F); the time required to complete 30 trials of C3 and C4 increased significantly compared with that of C0 (*F*_4_,_149_ = 24.22, *p* < 0.001 and *p* < 0.001). With images varying from easy to difficult, four mice failed to complete 30 trials in 60 min during C3 and C4, although 24 out of 28 mice succeeded, indicating the difficulty represented by the five images was suitable for this test. In consequence, the images adopted in this study showed a good difficulty gradient, implying a successful pattern completion evaluation paradigm. Combined with results in section “Establishment of paradigm to measure pattern separation,” we developed a method based on the touchscreen, which can measure pattern separation and pattern completion within the same apparatus.

## Discussion

Pattern separation and pattern completion are two important neural computational processes playing a critical role in episodic memory accuracy, and they contribute to cognitive impairments associated with neurodegenerative diseases. The existing paradigms [not including trial unique non-matching to location (TUNL) and location discrimination (LD)] used to measure pattern separation and pattern completion are confronted with many disadvantages, such as inadequate motivation, limited evaluation index, and stress from the environment. Besides this, in human beings, pattern separation and pattern completion could be evaluated in the same apparatus (Vieweg et al., 2019; Ngo et al., 2021), but it was never reported in rodents, which limited the mechanism and effect of cognition-enhancing drug research. In the past decade, the touchscreen apparatus had provided a new method to study cognition and related diseases, among which the pairwise discrimination task is not only moderate-difficult but also easy to operate (Bussey et al., 2012). Inspired by the object pattern separation paradigm and pattern completion paradigm derived from the Morris Water Maze task mentioned above, we established a method that can measure pattern separation and pattern completion based on the touchscreen pairwise discrimination task according to their definitions. In addition, the moderate-difficulty image in two paradigms, such as S2 and C2, enables bidirectional evaluation, that is, to evaluate the effect on promoting or damaging pattern separation and pattern completion.

The objects used in paradigms like the object recognition task must be attractive enough to ensure the animals are willing to explore and learn them (Burke et al., 2010; Leger et al., 2013); similarly, the images used in this paradigm must be moderate-difficult to ensure that most of the mice could complete the specified number of trials within a limited time. In this study, only three mice failed to complete 30 trials in 60 min during S3 and S4, and four mice failed during C3 and C4, indicating the images used in these paradigms are suitable for pattern separation and pattern completion evaluation. Among the five tests in pattern separation and pattern completion, there was no difference in the number of touches between the left and right screens during ITI (Supplementary Figures 1A, 3A), and the time to collect reward showed no significant difference whether the correct image appeared on the left or right screen (Supplementary Figures 1B, 3B), indicating the mice had no preference for left–right positions. Additionally, as the difficulty increased, there was no significant difference in the correct reward latency among the five groups both in pattern separation and pattern completion evaluation paradigms (Supplementary Figures 2, 4), implying the motivation of mice remained unaffected by task difficulty. No preference for position and unaffected motivation guaranteed the validity of the indices adopted in this study and the feasibility of the experiments.

The essence of pattern separation is to distinguish the correct information among similar ones; therefore, the more similar the information is, the worse the performance is. From S0 to S4, with the increasing similarity between the correct image and the incorrect one, the mice performed progressively worse, manifested as decreasing correct percentage, increasing correction trials and time, and presenting a good difficulty gradient change. The mice were re-baselined with pairwise discrimination task before pattern completion paradigm on 34th–36th days to guarantee they still remembered images “Marble” and “Fan.” The correct percentage of these 3 days were 85.0, 89.3, and 91.1%, respectively (Supplementary Figure 5), indicating the mice were qualified and ready for the next testing. Likewise, the essence of pattern completion is to retrieve intact memory according to partial or degraded cues; the more the cues information is, the better the performance of mice in pattern completion is. From C0 to C4, with the decreasing number of cues contained in the correct image, the mice performed progressively worse, manifested as decreasing correct percentage, increasing correction trials and time, and presenting a good difficulty gradient change. Besides this, multiple comparisons between S1–S4 and C1–C4 also indicated a great gradient change (Supplementary Tables 1, 2). As for the two paradigms, every index in each block also presented a good difficulty gradient change providing opportunities to track the dynamic changes in the performance of mice. Indices in each block may provide extra information, for example, the time required to complete the task could not reflect the difficulty gradient between C0 and C2 (Figure 4C), whereas the data in Block 3 (Figure 4F) could achieve that. In brief, the results showed that behavioral paradigms established in this study are suitable to measure pattern separation and pattern completion. We confirmed that the ability to discriminate similar information and the ability to retrieve the whole information according to partial cues can be indeed measured with paradigms in this study.

The paradigms established in this study are highly practical, efficient, and convenient, mainly reflected in the following aspects. (1) Paradigms for human beings are required to discriminate similar images *via* visual inputs, and by the same token (Riphagen et al., 2020; Li et al., 2021), we imitate them to develop paradigms for mice by displaying similar images on the touchscreen. (2) Bowen et al. (2020) and Miendlarzewska et al. (2016) believed that food restriction could promote task motivation. Mice have more exploratory motivation due to reasonable food restrictions in this study compared with paradigms based on natural curiosity. (3) Pattern separation and pattern completion can be measured on the same equipment with two basic images, which produces more reliable and comparable results. (4) Compared with the commonly used preference index [(T_novel_ − T_familiar_)/(T_novel_ + T_familiar_)] in the object recognition task (van Hagen et al., 2015), touchscreen-based paradigms have more metrics: correct percentage, number of correction trials, the total time required to complete the specified trials, and so on. Furthermore, we can obtain some more detailed results as 10 trials were divided into a block by the ABET II Touch software, by which the dynamics of mice performance were provided. (5) Well-trained mice can be tested repeatedly, avoid wasting animals, and enable contrasting themselves. (6) In paradigms derived from the object recognition task, sufficient arousal and intact locomotion are essential, there are minimal navigation and locomotion requirements within the touchscreen, though food restriction may reduce spontaneous locomotion (Moscarello et al., 2009). (7) The touchscreen apparatus is enclosed and driven automatically by software, avoiding stress from light, noise, odor, and experimenter. (8) Paradigms based on the touchscreen can be combined with other experimental techniques, such as electrophysiology (Marquardt et al., 2021a,b).

Though the paradigms we established were suitable for pattern separation and pattern completion evaluation, there still remained some limitations awaiting further discussion. (1) Compared with paradigms derived from the object recognition task, these two paradigms based on the touchscreen were suitable for pharmacodynamic evaluation and mechanism study, but not suitable for massive drug screening because too many compounds and groups are involved. (2) Some medicines or experimental treatments may disturb task motivation by affecting appetite or interacting with food, thus causing a bias toward the results.

As estrus cycle may cause performance variability in female mice (Frick and Berger-Sweeney, 2001; Meziane et al., 2007), only male mice were used in this study. However, the exclusion of the female mice is of serious concern as it is essential to the utility of this paradigm. Therefore, we will compare male and female mice to observe the effects of sex and sex hormone on pattern separation/completion based on this method in the future. There is no doubt that mice of different strains perform differently in the pairwise discrimination task (Oomen et al., 2013; Turner et al., 2017), unless some strains perform great in S4 and C4 (difficult) or poor in S0 and C0 (easy), the paradigms established in this study can be applied to most strains, but not all of them, which awaits further validation. These paradigms will be applied to other strains in further study, and hope they would be applied or modified by other researchers in their studies.

The method in this study and TUNL were both derived from the touchscreen apparatus, although they shared something in common, they were actually different. Both these two paradigms could avoid ceiling and floor effects as different difficulty stimuli were provided. In addition to pretraining, it usually takes at least 45–50 days for rats to complete the TUNL paradigm (Scott et al., 2021), but only 33 days for mice in the method established in this study. Besides this, TUNL was initially developed only for rats, as mice performed poorly (Oomen et al., 2013). More training sessions and higher difficulty make TUNL more time-consuming and less efficient compared with paradigms in this study. This unique method is capable of both pattern separation and pattern completion evaluation. In principle, TUNL is used to tax spatial pattern separation based on the perception of distance, and the paradigm in this study is used to tax visual pattern separation based on the holistic image recognition. Paradigms in this study and TUNL can complement each other, which is of great significance to the comparative study of the mechanisms and characteristics of pattern separation of different kinds.

Paradigms established in this study based on the touchscreen pairwise discrimination task showed a gradual difficulty gradient with images varying from easy to difficult, indicating they were qualified paradigms to assess pattern separation and pattern completion. This would enable the paradigms useful for investigating the mechanisms underlying episodic memory or neurodegenerative diseases in which pattern separation/pattern completion are impaired.

## Conclusion

The construct validity of these two paradigms designed to measure pattern separation and pattern completion in mice is based on their definition. Once the mice learned the basic pairwise discrimination task, pattern separation and pattern completion can be measured within the same apparatus, which facilitates the comparability of the two processes. Strong motivation, animal reuse, multiple evaluation indices, high efficiency, fewer locomotion requirements, and stress-free provide advantages of these paradigms. Owing to the gradual changing difficulty, both impairment and enhancement of pattern separation or pattern completion can be easily measured. To further broaden the applicability of these two paradigms based on touchscreen pairwise discrimination task, other strains of mice will be validated in the future.

## Data availability statement

The original contributions presented in this study are included in the article/Supplementary material, further inquiries can be directed to the corresponding authors.

## Ethics statement

The animal study was reviewed and approved by the Institute of Animal Care and Use Committee (IACUC) of the National Beijing Center Drug Safety Evaluation and Research (NBCDSER).

## Author contributions

WZ, YH, and YZ designed the study and were responsible for editing the manuscript. HW wrote the manuscript and executed three-fourths of the experiments and statistical analysis. NS executed one-fourth. XW and JH jointly completed data curation. All authors read and agreed to the published version of the manuscript.

## Footnotes

1
https://github.com/1996haowang/images-for-PS-and-PC.git
