# Supplementary material for: A touchscreen-based paradigm to measure visual pattern separation and pattern completion in mice
Source: Front Neurosci. 2022 Aug 24;16:947742. doi: 10.3389/fnins.2022.947742 (PMC9449699; doi:10.3389/fnins.2022.947742)
Supplement: Supplementary file 1 [file Data_Sheet_1.docx]

Supplementary Material


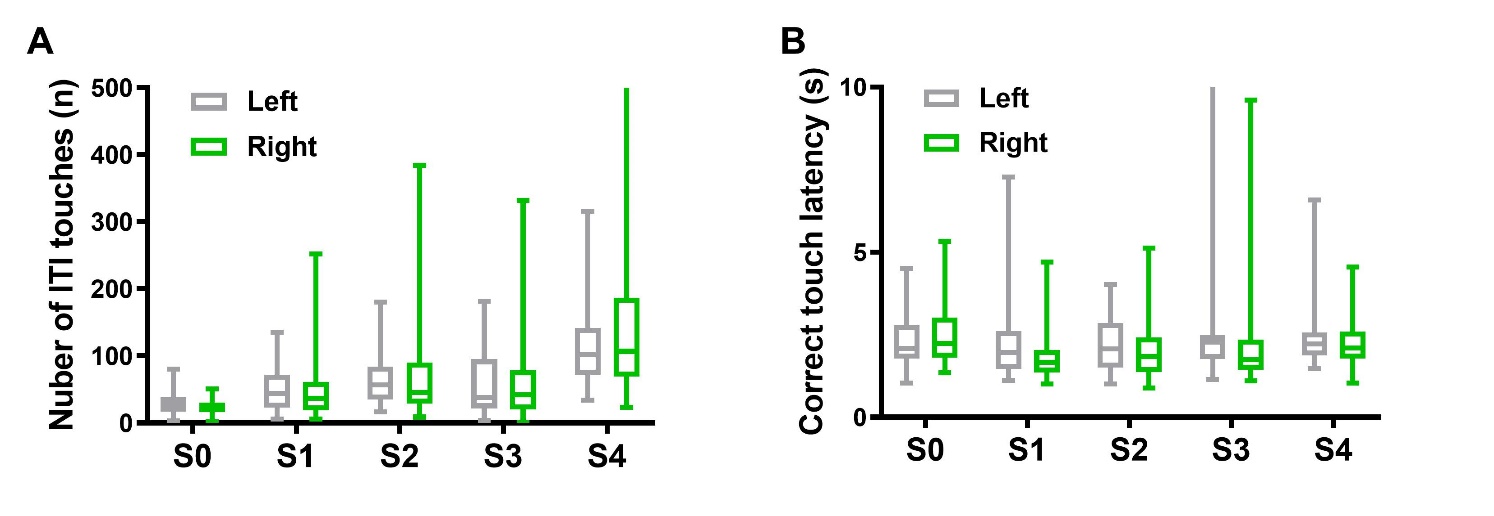


**Supplementary Figure 1.** The preference for left-right positions of mice in pattern separation evaluation paradigm. (A) Number of touches during Intertrial Interval (ITI). (B) Correct touch latency when the correct image appeared on the left or right position. Data is presented as the mean ± SD (n = 30-31).


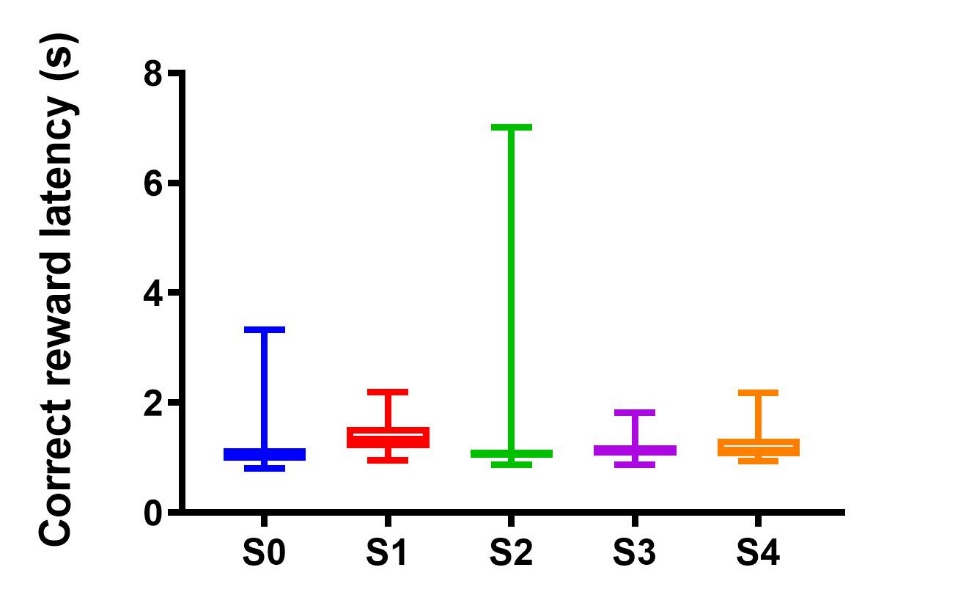


**Supplementary Figure 2.** The time required to collect the reward when the mice complete a trial correctly in pattern separation evaluation paradigm. Data is presented as the mean ± SD (n = 30-31).


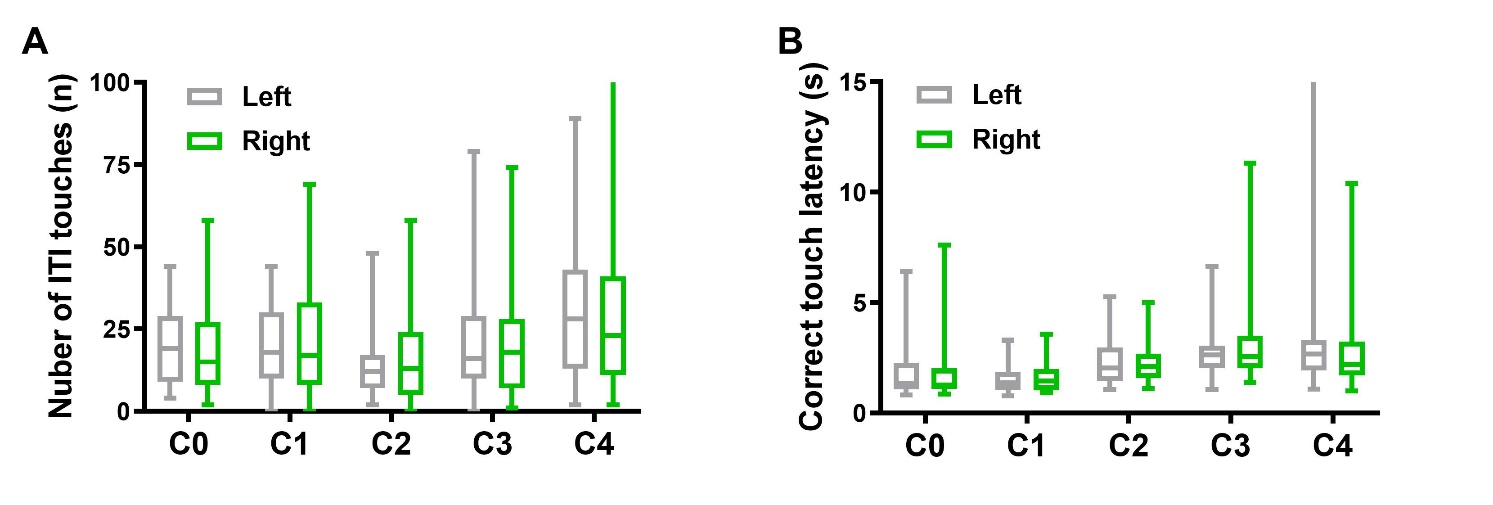


**Supplementary Figure 3.** The preference for left-right positions of mice in pattern completion evaluation paradigm. (A) Number of touches during Intertrial Interval (ITI). (B) Correct touch latency when the correct image appeared on the left or right position. Data is presented as the mean ± SD (n = 26-28).


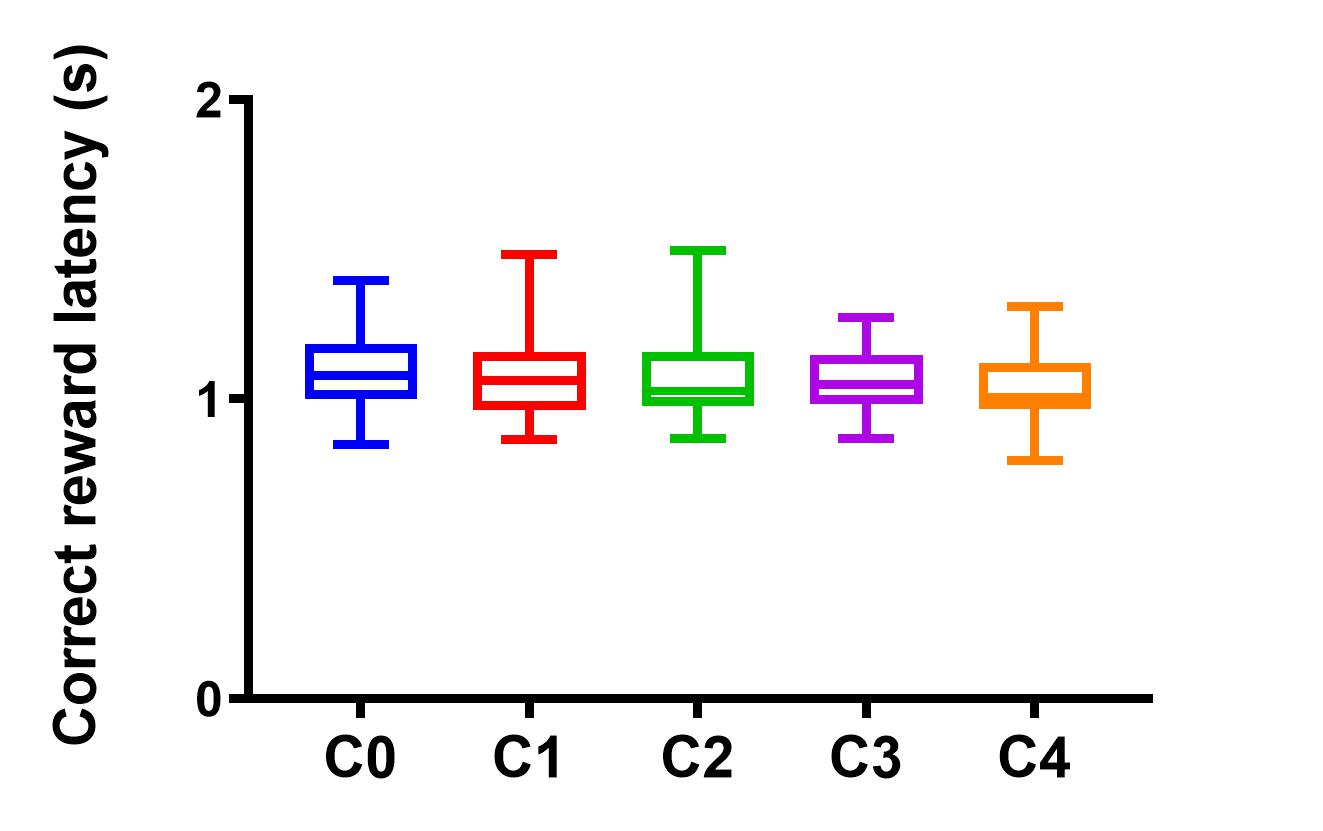


**Supplementary Figure 4.** The time required to collect the reward when the mice complete a trial correctly in pattern completion evaluation paradigm. Data is presented as the mean ± SD (n =26-28).


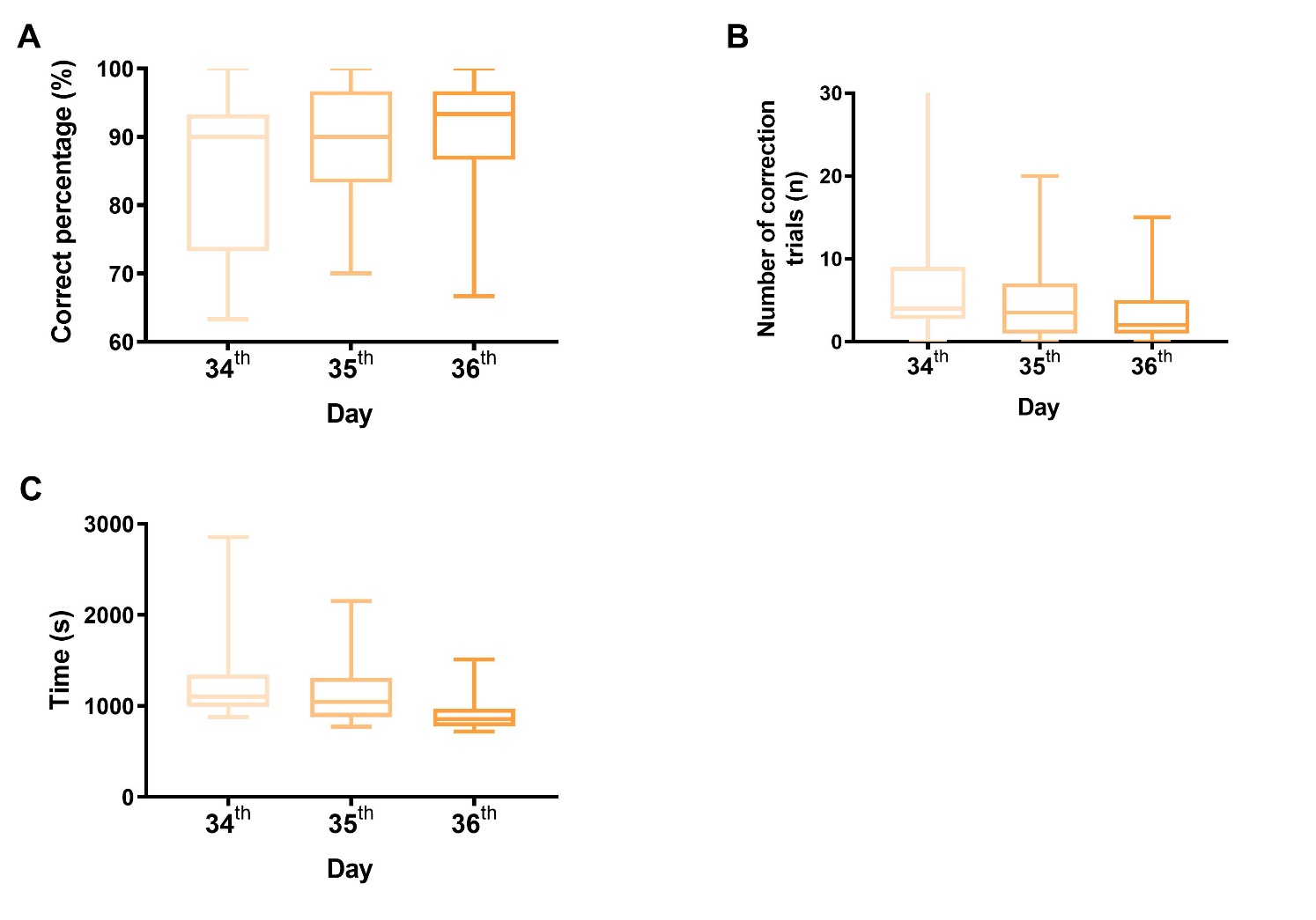


**Supplementary Figure 5.** Re-baselining training between pattern separation and pattern completion test. Data is presented as the mean ± SD (n = 30-31).

**Supplementary table1** Multiple comparisons between S1-S4 of mice

|  | **Correct percentage** | | | **Correction trials** | | | **Time** | | |
| --- | --- | --- | --- | --- | --- | --- | --- | --- | --- |
|  | **S2** | **S3** | **S4** | **S2** | **S3** | **S4** | **S2** | **S3** | **S4** |
| **S1** | *p*=0.0001 | *p*<0.0001 | *p*<0.0001 | *p*=0.0398 | *p*=0.0001 | *p*<0.0001 | *p*=0.0161 | *p*=0.0001 | *p*<0.0001 |
| **S2** |  | *p*=0.0636 | *p*<0.0001 |  | *p*=0.2815 | *P*<0.0001 |  | *p*=0.4990 | *p*<0.0001 |
| **S3** |  |  | *p*=0.0027 |  |  | *p*<0.0001 |  |  | *p*<0.0001 |

**Supplementary table 2** Multiple comparisons between C1-C4 of mice

|  | **Correct percentage** | | | **Correction trials** | | | **Time** | | |
| --- | --- | --- | --- | --- | --- | --- | --- | --- | --- |
|  | **C2** | **C3** | **C4** | **C2** | **C3** | **C4** | **C2** | **C3** | **C4** |
| **C1** | *p*=0.2086 | *p*<0.0001 | *p*<0.0001 | *p*=0.5574 | *p<0.0001* | *p*<0.0001 | *p*=0.6988 | *p*<0.0001 | *p*<0.0001 |
| **C2** |  | *p*<0.0001 | *p*<0.0001 |  | *p*=0.0003 | *p*<0.0001 |  | *p*=0.0012 | *p*<0.0001 |
| **C3** |  |  | *p*=0.0033 |  |  | *P*=0.0005 |  |  | *p*=0.2420 |
